# Grisel’s syndrome: a case report on this rare pediatric disease and its anesthetic challenges

**DOI:** 10.1186/s12871-020-01176-7

**Published:** 2020-09-30

**Authors:** Kavya N. Reddy, Shabaaz M. Baig, Meenu Batra, Kevin Colodner, Uchenna Madubuko, Anna Korban, Shridevi Pandya Shah

**Affiliations:** 1 Saint Louis University, St. Louis, USA; 2 Department of Anesthesiology, Rutgers New Jersey Medical School, 185 South Orange Ave, MSB-E 577, Newark, NJ 07103 USA; 3 University of Maryland, College Park, USA; 4 New York Medical College, Valhalla, USA

**Keywords:** Grisel’s syndrome, Atlantoaxial subluxation (AAS), Pediatric anesthesia, Airway management, Positioning, Cervical spine

## Abstract

**Background:**

Grisel’s syndrome is a non-traumatic atlantoaxial subluxation associated with inflammatory conditions of the head and neck, which occurs primarily in children. Increased flexibility of the ligaments during inflammation is implicated in the pathogenesis of the subluxation between the axis and atlas. The potential sequelae may be severe, and early diagnosis and treatment of Grisel’s syndrome can prevent tragic outcomes.

**Case presentation:**

We present a case of torticollis in an 8-year-old child. She had a two-week history of a streptococcal throat infection. The patient was treated with several different methods of conservative care, including muscle relaxation, cervical halter traction, and Halo application. However, the torticollis persisted. The patient then required surgical correction involving cervical spine fusion. She had no complications and experienced no reoccurrence of the torticollis to date.

**Conclusion:**

Grisel’s syndrome is a pathology for which conservative management is successful in most cases. Cases requiring surgical intervention are rarely documented in the literature. Our case is significant, as in spite of aggressive conservative management, the patient required surgical correction. Patients requiring surgical management of Grisel’s syndrome may require additional anesthetic exposure for diagnostic interventions like magnetic resonance imaging or neck manipulations for closed reduction. We discuss the features of Grisel’s syndrome and specific anesthetic management considerations for procedures such as magnetic resonance imaging, application of cervical traction, and surgical correction of torticollis.

## Background

Nontraumatic atlantoaxial subluxation (AAS) is a rare complication of upper respiratory tract infections and otolaryngologic surgeries first described by Sir Charles Bell in 1830 in a patient with syphilis, pharyngitis, and lethal spinal compression. A century later, a French physician, Grisel, described two cases of pharyngitis and nontraumatic AAS and named it Grisel’s syndrome [1]. Grisel’s syndrome refers only to non-traumatic AAS, and most commonly occurs after an upper respiratory infection, though some unusual causes include mumps, tuberculosis, and Kawasaki disease [2]. It may also be seen after certain otolaryngologic operations such as adenoidectomies, tonsillectomies, and ear surgeries.

Grisel’s syndrome is most often seen in children but may also be seen in adults. Originally, Grisel theorized that the subluxation occurs secondary to muscular spasm. The most recent explanation is the two-hit hypothesis, which posits that hyperemia following infection or trauma leads to decalcification of the anterior arch of C1 and laxity of the transverse ligament [3]*.* Fielding has characterized four different types of rotatory AAS (see Table 1) [4].

**Table 1:** Types of Rotary Atlantoaxial Subluxation (AAS)

Type I	The atlas is rotated on the odontoid, and there is no anterior displacement of the atlas
Type II	The atlas is rotated on one lateral articular process with 3 to 5 mm of anterior atlas displacement
Type III	Rotation of the atlas with an anterior displacement greater than 5 mm
Type IV	Rotation and posterior displacement of the atlas

Types I and II are most often seen without any neurological deficits and are typically resolved conservatively, with oral non-steroidal anti-inflammatory drugs (NSAIDs), antibiotics, physical therapy, and the collar. Types III and IV are more severe and are associated with neurological deficits and serious complications and are managed with surgical fusion. The most definitive imaging modalities for AAS are computed tomography (CT) and magnetic resonance imaging (MRI) studies.

In our study, we review the literature published so far, which indicates the most common etiologies, diagnostic modalities, and management of these patients. There is, however, a paucity of literature regarding the anesthetic management of this pathology. We present the case of our patient with Grisel’s syndrome who failed conservative management and had to undergo surgical fixation with a discussion on the anesthetic management and challenges we faced in this case.

Informed consent was obtained from the patient’s parents for this clinical case report.

## Case presentation

We report a case of an 8-year-old female with a 2-week history of recent streptococcal throat infection who presented to the emergency room with torticollis. She was treated conservatively with antibiotics, muscle relaxation, and physiotherapy. Neither an otolaryngology consult nor radiological tests were ordered. A month later, her torticollis persisted, for which she received a CT scan. It showed a C1-C2 AAS, which was consistent with a diagnosis of Grisel’s syndrome. At this point, conservative management was continued with cervical halter traction of 5 lbs., which was subsequently increased to 8 lbs. during her month-long stay in the pediatric intensive care unit (PICU). Post manual reduction, she had full range of motion of her neck, and CT showed complete resolution of subluxation. However, she returned with persistent torticollis 3 months later. An MRI at this time showed C1-C2 rotatory subluxation with associated narrowing of the cervico-medullary junction. She underwent Halo application under anesthesia, which was uneventful. After a week, however, the patient showed no improvement. The next step was C1-C2 cervical fusion under general anesthesia in the operating room.

**Fig. 1:**
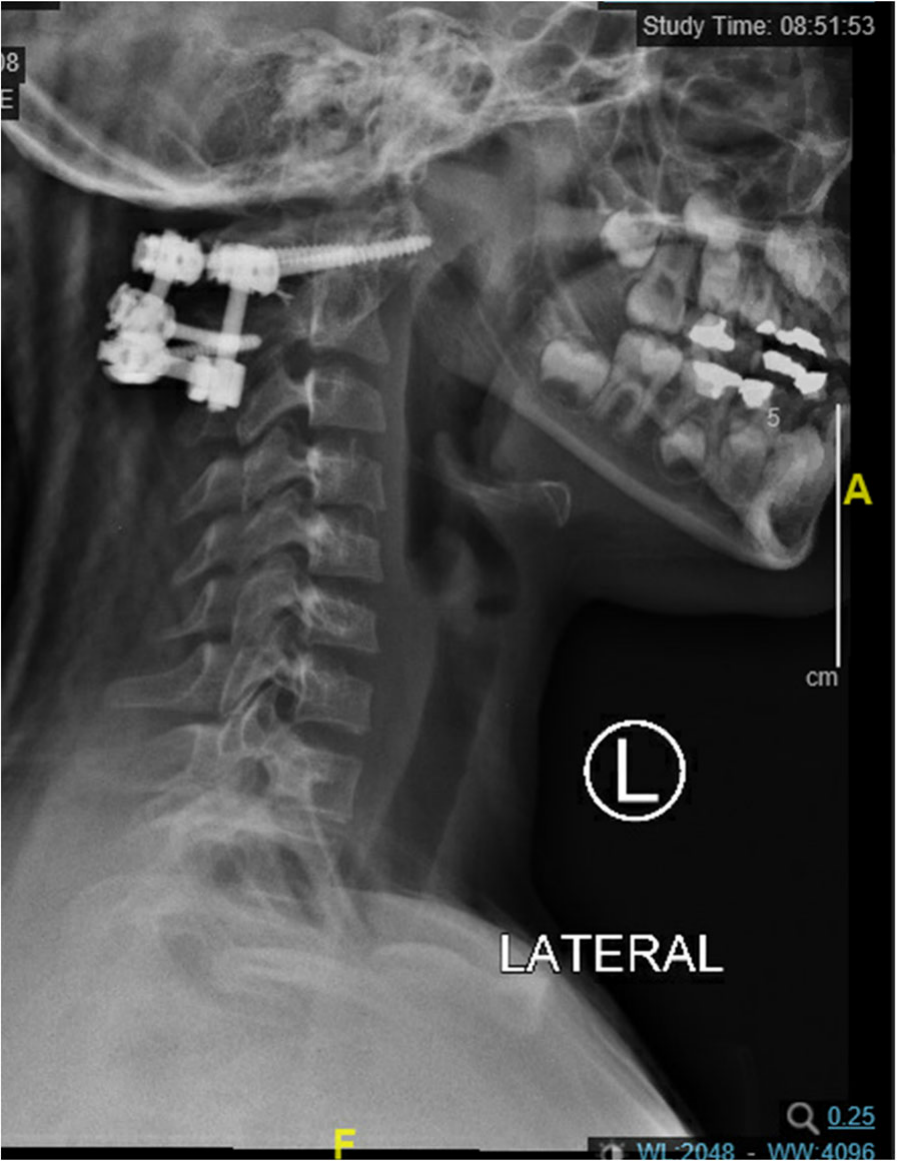
lateral view of C1-C2 postsurgical fixation

The patient was brought to the operating room and standard monitors were placed. Total intravenous anesthesia was requested by the surgeon as motor and somatosensory evoked potentials would be monitored during surgery. The patient was induced with 200 ml of propofol, 100 mcg of fentanyl, and 2 mg of versed through a preexisting intravenous (IV) line. No muscle relaxants or inhalational agents were used during induction or maintenance of anesthesia. The patient was intubated endotracheally using the video laryngoscope while maintaining inline stabilization of the neck and spinal cord. Patient had been intubated with use of conventional direct laryngoscopy during her previous anesthetic. And so fiberoptic bronchoscope was not required. A second large bore IV and arterial line were placed after intubation. Total intravenous anesthesia (TIVA) was used for maintenance with an infusion of 0.1–0.2 mcg/k/min remifentanil and 100–120 mcg/kg/min propofol as tolerated intraoperatively. Somatosensory evoked potentials and motor evoked potentials remained unchanged throughout the surgery. Duration of surgery was about 10 h. The estimated blood loss was one liter. The patient received 2 l of crystalloids, 250 cc of albumin, one unit of packed red blood cells, one unit of fresh frozen plasma, and 500 cc of cell saver. The patient had a foley placed prior to the surgery which showed a urine output of 800 ml. Given that the length of surgery was 10 h, the major fluid shift, and the need for immobilization, the patient was left intubated after surgery. The propofol infusion was continued postoperatively for sedation and she was transferred to the care of the PICU. She was extubated the next day. A follow-up CT scan showed a complete resolution of subluxation. Based upon follow up imaging (see Fig. 1), there were no adverse or unexpected outcomes.

## Discussion

A PubMed search was performed using the phrases “Grisel’s syndrome,” “spontaneous AAS,” and “ENT surgery complications.” We made an extraction form focusing on the etiology, diagnostic modalities, treatment received (conservative or surgical), and follow-up outcomes. Single case reports and case series of both pediatric and adult cases were included. We identified 115 papers, of which 15 papers were excluded as they were not in English. We were able to analyze 100 papers evaluated by three independent reviewers. After an analysis of those 100 papers, we found 80 papers that met the inclusion criteria of nontraumatic AAS, Grisel’s syndrome, and torticollis. Of those 80 papers included in the study, 62 were case reports (62 cases), 14 were case series (37 cases), and 4 were retrospective reviews (1 case report, 3 case series (20 cases)), making a total of 119 cases for our purposes. Out of these 119 cases, 55% described infection as the causative factor, and 45% had a post-surgical cause as the etiology. Adenoidectomy (30%), oto-surgery (15%), adenotonsillectomy (13%), and tympanoplasty (7%) were the most common surgical procedures established as the primary cause of AAS in the category of post-surgical AAS.

Diagnosis of the disease process was by CT scan (55%) followed by X-ray only (19%), X-ray diagnosis with CT confirmation (18%), and direct MRI confirmation (8%). In our analysis, treatment for cases of infectious origin were successful mainly through conservative methods such as antibiotics, NSAIDs, cervical collars, or Halo application. Surgical fusion was employed in 17% of cases due to failed conservative treatment. In post-surgical patients, conservative treatment was successful in 93% of cases. The remainder had to undergo surgical fusion. The most likely reason for an increased failure of conservative management and the necessity of surgical fusion in the cohort of patients with an infectious etiology is the duration of symptoms and delayed diagnosis.

Grisel’s syndrome is a diagnosis of exclusion. Differential diagnoses such as traumatic head posture and developmental torticollis that must be ruled out first. Congenital conditions that involve ligamentous laxities, such as Marfan syndrome and Down syndrome, are more susceptible to AAS [5]. Rare causes of AAS include tuberculosis [6] and Kawasaki disease [7]. Case reports have linked Grisel’s to the use of monopolar suction electrocautery in adenoidectomy for bleeding control [5]. Karkos et al. reported 96 cases, of which 48% were due to infections [8]. Bocciolini et al. described 100 cases, of which 77 cases were caused by infection [9]. CT with 3D reconstruction proved to be an excellent method of documenting the presence and degree of AAS [5].

Neurological complications are infrequent but can be devastating and are seen in 15% of cases. They can range from radiculopathy to quadriplegia and death from respiratory failure due to medullary compression. Diagnosis of AAS should be based on a high degree of clinical suspicion as early detection and treatment are critical factors in preventing severe neurological complications. Post-surgical neck pain and torticollis are early signs of AAS and should not merely be attributed to the usual post-surgical pain and malaise.

Administration of anesthesia, especially airway management, in these patients presents a significant challenge. This is due to the abnormal laxity/inflammatory distention of cervical ligaments. These patients are at high risk of neurological injury during laryngoscopy, and tracheal intubation as a head extension or any sudden movements of the cervical spine may cause subluxation of the atlas over the axis, resulting in spinal cord compression. Therefore, one needs to maintain inline cervical stabilization, preferably using a video laryngoscope, and be prepared with a difficult airway cart including need for fiberoptic bronchoscope [10]. Gupta et al. also suggests using fentanyl for analgesia to prevent the general anesthetic complications of respiratory depression and myocardial depression [10]. Postoperatively patients with AAS must be carefully monitored for signs of neurological complications. Keeping the patient immobilized postoperatively and under close watch in the intensive care unit is one strategy to prevent these issues from arising. When patients require continued ventilation after surgery, consideration should be given to allow them to awaken after the procedure, to test for signs of myelopathy.

Positioning for otolaryngologic procedures such as tonsillectomies, direct laryngoscopies, or ear procedures also deserves deliberation. As positioning is an important part of anesthetic management, being aware of the optimal position for a patient with AAS is critical. This will help alleviate the chances of postoperative neurological complications commonly associated with AAS. For myringotomies, it is recommended to strap the patient to the operating room table securely, place supports alongside the head, and roll the table side to side, rather than turning the head. For airway procedures that usually involve head extension, it is crucial to communicate with the surgeon preoperatively about minimizing head and neck movement. It may be possible for the surgeon to perform the laryngoscopy or tonsillectomy without using suspension.

For urgent surgeries, the patient should be treated with cervical spine precautions. An unstable cervical spine will require neurological and electrophysiological monitoring. TIVA and delivery of anesthesia to patients with cervical traction and halo outside the operating room in MRI and CT suites can also be considered.

Grisel’s syndrome remains a rare disease with little in the literature regarding its implications in anesthesia. If we review the literature, we may be able to find principles of anesthetic management for similar types of spine surgery. However, anesthetic management of Grisel’s syndrome has its unique implications. Challenges include, but are not limited to, the usage of TIVA, airway management, positioning, and anesthesia outside of the operating room. The practicing anesthesiologist would be well served by being aware of this disease and its management to ensure the safety and health of their patients.

## Data Availability

The datasets used and/or analyzed during the current study are available from the corresponding author on reasonable request.

## Abbreviations

AAS: Atlantoaxial Subluxation
NSAIDs: Non-steroidal Inflammatory Drugs
CT: Computed Tomography
MRI: Magnetic Resonance Imaging
IV: Intravenous
PICU: Pediatric Intensive Care Unit
TIVA: Total Intravenous Anesthesia
